# The Dialectical Relationship Between Burnout and Work Engagement: A Network Approach

**DOI:** 10.1002/smi.3514

**Published:** 2024-12-04

**Authors:** Marius D. Hafstad, Omid V. Ebrahimi, Knut Inge Fostervold

**Affiliations:** ^1^ Department of Psychology University of Oslo Oslo Norway; ^2^ Department of Experimental Psychology University of Oxford Oxford UK; ^3^ Research Institute Modum Bad Psychiatric Hospital Vikersund Norway

**Keywords:** burnout, network analysis, psychological well‐being, work engagement

## Abstract

Based on previous empirical evidence, scholars have argued for a dialectical relationship between burnout and work engagement. However, these conclusions largely rely on latent variable models, which have inherent limitations due to strong hierarchical assumptions about data. As a result, previous findings should be considered preliminary indications rather than conclusive evidence. In this study, we introduce network analysis to investigate the relationship between burnout and work engagement. We demonstrate its utility by comparing the fit indices of the network model and three factor models in a cross‐national sample with 2469 employees from Norway and 879 employees from the US and UK. Based on theory and fit indices, we conclude that the network model was preferred in both samples. Using this model, we uncovered *positive* relationships between two work engagement variables and the exhaustion component of burnout. Theoretically, this study broadens our understanding and show support for the dialectical relationship between burnout and work engagement. Furthermore, by introducing the network model to empirical research, we provide a novel approach that contribute to nuance and ideas for research on burnout and work engagement. Practically, our results offer insightful data on possible points for intervention between burnout and work engagement variables.

## 1 Introduction

In recent decades, there has been considerable interest in the concepts of burnout and work engagement, two subjective well‐being constructs that have been linked to various outcomes such as performance, health, and citizenship behaviours (Ahola 2007; Halbesleben and Wheeler 2008; Harter, Schmidt, and Hayes 2002). Burnout can be characterised as a combination of chronic exhaustion and cynical attitude towards work (Demerouti et al. 2021), while work engagement can be characterised by vigour, dedication and absorption in one's work (Schaufeli et al. 2002). However, alongside their popularity, several researchers have questioned their empirical distinctiveness which has prompted a wide range of studies to better understand the nature of their empirical relationship (e.g., Cole et al. 2012; Taris, Ybema, and van Beek 2017; Trógolo et al. 2020).

Currently the understanding is that like other subjective well‐being constructs (e.g., positive and negative affect; Diener, Lucas, and Oishi et al. 2018), burnout and work engagement are inversely related but exhibit a degree of independence (for an overview see Schaufeli and De Witte 2017). In other words, they are different, but they also share certain features. Leon, Halbesleben, and Paustian‐Underdahl (2015) argue that this empirical relationship can be explained by dialectical theory. The theory aim to explain the existence of contradictions (paired opposites; Baxter 1990) when two seemingly opposing constructs are mutually dependent and negatively related (Baxter 1990). According to dialectical theory, a comprehensive understanding of burnout and work engagement is achieved in context of the contradicting phenomena. Due to their influence on each other, burnout and work engagement act on, and work against, the other (Baxter 1990; Leon, Halbesleben, and Paustian‐Underdahl 2015). This means that an employee can experience the two states simultaneously, not at all, or one dominating the other, all of which can generate unique outcomes. For example, by simultaneously experiencing burnout and work engagement, employees may be prone to working while sick (i.e., presenteeism; Leon, Halbesleben, and Paustian‐Underdahl 2015).

However, while Leon, Halbesleben, and Paustian‐Underdahl (2015) argue that network analysis is well‐suited for capturing the dialectical nature of the relationship, previous research has analysed it using latent variable models. This is problematic because one key limitation with the latent variable model is its violations of direct causal effects, semantic overlap, and reciprocal interactions between indicators or items (Borsboom 2008; Cramer et al. 2010, 2012). This, in turn, obscures the ability to truly understand the dialectical associations between items. Thus, prior studies provides indications but not conclusive support for the dialectical theory of burnout and work engagement.

Explained in more detail later, the network approach diverges from the traditional latent understanding of phenomena by assuming that observed variables does not reflect an underlying latent construct; the variables and their connections *is* the construct and is tied to the operationalisation and measurement of the phenomena (Burger et al. 2022; Fried 2017; Malgaroli, Calderon, and Bonanno 2021; Rodebaugh et al. 2018). Recent research by Trógolo et al. (2020) have taken a step towards adopting a more comprehensive model by using the bifactor model to represent the burnout‐work engagement relationship. However, the use of the bifactor model raises several concerns, including evidence of overfitting, unstable parameter estimates, and statistical bias in favour of bifactor models, even when other simpler models fit similarly well (Morgan et al. 2015; Murray and Johnson 2013; Watts, Poore, and Waldman 2019).

Based on this, the aim of this paper is to introduce a novel and complementary perspective to the current burnout and work engagement debate (Trógolo et al. 2020) by examining the relationship using a network analytic framework. We agree that ‘considering burnout and engagement complete opposites (or a single construct) is inadequate’ and an ‘incomplete view’ (Leon, Halbesleben, and Paustian‐Underdahl 2015, p. 92). By representing the constructs as part of a system that assumes reciprocal relationships between variables, we uncover previously unknown associations between variables. To evidence the benefits of a network perspective, we use three samples collected from the US, the UK, and Norway to examine and compare fit measures between one network model and three latent variable models. By testing the relationship across countries, we test the cross‐cultural validity of these concepts and showcase invariant relationships (Dong and Dumas 2020; Leitgöb et al. 2023).

We make three contributions that provide theoretical, empirical and practical implications. Theoretically, we provide the first initial test and model that accounts for the possibility of a dialectical relationship. We reveal results that add to our understanding of how a dialectical relationship of contradiction can exist between burnout and work engagement (i.e., they share an intricate relationship). Also, by testing the network model across samples of employees from the US, the UK, and Norway, we provide evidence for a dialectical relationship despite cultural variation.

Empirically, we provide a new approach to the relationship between burnout and work engagement (Leon, Halbesleben, and Paustian‐Underdahl 2015). Also, we provide a novel approach for future research to examine theories that include burnout and work engagement, such as Job Demands‐Resources theory (Bakker, Demerouti, and Sanz‐Vergel 2023; Demerouti et al. 2000) and the Effort‐Recovery model (Fostervold and Watten 2022; Meijman and Mulder 1998). Unlike latent variable modelling, our approach highlights the relationships that is unique to each pair of variables (Epskamp et al. 2016), which is important because variables can vary in the amount of variance they contain (Richardson, Simmering, and Sturman 2009; Spector et al. 2019).

Practically, by introducing the network approach to data, we introduce a technique that could provide practical knowledge and possibilities for intervention research on burnout and work engagement (Demerouti et al. 2021). The field of work and organisational psychology has been accused of distancing itself from practice (Arnold, Dries, and Gabriel 2021), leading to limited implications for leaders and organisations (Tourish 2020). When matched with the appropriate design, network approaches have been applied to guide personalised, optimal treatment strategies (Bak et al. 2016; Fisher et al. 2017; Lutz et al. 2018; Ryan and Hamaker 2022).

In the next sections, we introduce the history and relationship between burnout and work engagement and depict previous results that have largely relied on what we refer to as ‘the continuum’‐model or ‘the distinct but inversely related’‐model. We then introduce, explain, and highlight benefits of network theory for research on the dialectical theory of burnout and work engagement. Finally, to show the importance of a network perspective, we analyse and compare fit indices of three latent factor models and one network model, in two separate estimations. Finally, we discuss the theoretical, empirical, and practical implications.

### 1.1 Burnout and Work Engagement

Burnout was originally defined by Maslach and Jackson (1981) as a work‐related stress syndrome characterised by emotional exhaustion, depersonalisation and reduced personal accomplishment (i.e., professional self‐efficacy; see Demerouti et al. (2021) for an overview). More recently, burnout has been revised to consist of the two core dimensions exhaustion and cynicism (Halbesleben and Demerouti 2005; Schaufeli, Enzmann, et al. 2017; Schaufeli and Taris 2005). Professional self‐efficacy (the evaluation of achievement and competence at work) has been suggested to be more a personality trait (Cordes and Dougherty 1993), an antecedent (Ventura, Salanova, and Llorens 2015), or a moderator of the job stressor‐burnout relationship (Gil‐Monte, García‐Juesas, and Hernández 2008).

Maslach and Leiter (1997) defined work engagement as the opposite of burnout ‐ a positive well‐being state characterised by a high level of energy and strong identification with one's work. According to the authors, burnout and work engagement represented opposing ends on an occupational well‐being spectrum. Consequently, a separate measure of work engagement was superfluous. Schaufeli and colleagues (Schaufeli and Bakker 2004; Schaufeli et al. 2002), on the other hand, argued that burnout and work engagement were conceptually opposites, yet distinct states, and work engagement could be operationalised as a three‐dimensional construct, consisting of vigour, dedication, and absorption.

Similarly to burnout, some authors (e.g., Innanen, Tolvanen, and Salmela‐Aro 2014) argue that vigour (the experience of high levels of energy) and dedication (a sense of meaningfulness and challenge) represent work engagements' two core dimensions (Llorens et al. 2007; Schaufeli and Bakker 2004). Absorption (the act of being deeply focused and finding it difficult to stop working) represent, in their view, a more independent construct. Other authors have pointed out that absorption may represent a consequence of work engagement (Salanova et al. 2003) or a non‐exclusive feature of work engagement (2008, 2008). Consequently, a few previous studies have excluded absorption when examining the burnout‐work engagement relationship. However, the theoretical as well as the empirical rationale for excluding absorption is questionable. In the case of excluding professional inefficacy from burnout, this was based on theoretical and empirical demonstrations. The case is not similar for work engagement which, based on the theoretical understanding and strong support for a unidimensional UWES scale, guide us to include absorption in this study (Schaufeli 2012; Schaufeli, Shimazu, et al. 2017; Seppälä et al. 2009).

#### 1.1.1 Previous Models of the Relationship Between Burnout and Work Engagement

Previous efforts to model the relationship between burnout and work engagement have either examined burnout and work engagement as two distinct, albeit related, constructs, or as two constructs on a subjective well‐being spectrum. Early research concluded that a model with correlated factors, assuming burnout and work engagement as different but related was preferred (Schaufeli and Bakker 2004; Schaufeli et al. 2002, 2006; Schaufeli, Taris and Van Rhenen 2008). For example, Schaufeli and Bakker (2004) compared a model where burnout and work engagement where separate yet correlated, and a model where burnout and work engagement were facets of a second‐order well‐being factor. They preferred the former because the second‐order well‐being model did not reach satisfactory fit. In the same study, Schaufeli and Bakker (2004) examined the nomological net of burnout and work engagement. Their analysis revealed that burnout and work engagement displayed different patterns of relationships with antecedents (e.g., job demands and resources) and outcomes (e.g., health outcomes and turnover) across four samples.

Other studies have documented a more complex and overlapping relationship between burnout and work engagement, suggesting a continuum model. For example, González‐Romá et al. (2006) found, in three samples of knowledge workers, that cynicism (burnout) and dedication (engagement) represents an identification continuum, while exhaustion (burnout) and vigour (engagement) represents an energy continuum. A similar study was carried out by Demerouti, Mostert, and Bakker (2010) who replicated the identification continuum but not the energy continuum in a sample of 528 construction workers. Later, Cole et al. (2012) carried out a meta‐analysis on more than 19 articles and a sample of over 14.000 individuals. They compared four different latent models and concluded that the model with cross‐loadings from the latent burnout construct to the work engagement variables were preferable. In their view, burnout and work engagement were mere opposites on a subjective well‐being spectrum.

More recent studies confirm the more complex and overlapping understanding of the relationship (Taris, Ybema, and van Beek 2017; Trógolo et al. 2020). For example, Taris, Ybema, and van Beek (2017) examined burnout and work engagement in a sample of 1535 Dutch police officers. They found that burnout and work engagement are highly overlapping, indicated by the correlations between core dimensions (−0.60 to 0.87) and the two latent variables (−0.75), yet they did not share a second‐order latent well‐being construct. Additionally, and in contrast to Schaufeli and Bakker (2004), their analysis of the nomological net showed that burnout and work engagement had highly similar correlations with other constructs.

In summary, previous studies seem to be weighted towards ‘the distinct yet negatively related’‐model. In other words, burnout and work engagement is somewhat inversely related and overlapping, yet with distinctive features. This brief review of a complex pattern of relationships reveals further, however, the close to exclusive reliance on latent variable models. In the next section, we describe why these results in essence reveal the potential for a network perspective. Briefly, we argue that network models can provide a complementary, yet a more congruent explanation for how the phenomena can be distinct yet inversely related.

### 1.2 It's a Matter of Perspective ‐ Network Theory's Philosophy, Concept, and Models

The network approach to understanding the emergence of psychological phenomena has had an impact in a diverse range of subfields of psychology, such as anxiety (Hoffart et al. 2023), attitudes (Dalege et al. 2016), addiction (Bereznowski et al. 2021), personality (Cramer et al. 2012) and intelligence (Kan, van der Maas, and Levine 2019). The network approach is based on the premise that observed variables, and their relations, are not assumed to reflect an underlying latent construct but are assumed to stem from a network of causally related variables (Borsboom and Cramer 2013). In other words, variables are mutually interacting, and can be reciprocally reinforcing, elements (Borsboom 2017; Ebrahimi 2023). For example, from this perspective, burnout is not an underlying latent construct that is reflected through the feeling of exhaustion or cynical attitudes towards work. It is rather a system of directly and indirectly interrelated variables that combine to produce the state of burnout. Consequently, the network analysis is tied to the operationalisation and measurement of the phenomena (Burger et al. 2022; Fried 2017; Malgaroli, Calderon, and Bonanno 2021; Rodebaugh et al. 2018).

In contrast, latent variable modelling assumes that variables, or indicators, are locally independent and exchangeable (e.g., Bollen 1989; Borsboom 2008; Borsboom, Mellenbergh, and Van Heerden 2003; Cramer et al. 2010). For example, local independence means that the variables in our study ‘I have become more cynical towards if my work contributes to something’ and ‘I am less enthusiastic about work’ have no impact on each other but is merely the result of an underlying, latent burnout syndrome. These two indicators can further be equally exchanged for a third variable that measures cynicism: ‘I have become less interested in my work’. Furthermore, adding the latter variable to the former variables will only contribute to increased reliability but no additional information. This assumption of local independence has generally led to critique of the latent variable model in psychology, and the latent variable model has found to have frequent violations of semantic overlap and direct causal and reciprocal interactions between indicators (Borsboom 2008; Cramer et al. 2010, 2012).

Thus, based on these problematic features, network analysis has been introduced to psychological phenomena, and could serve as a competing philosophical, theoretical, and empirical approach to the current understanding of burnout and engagement. The network approach represent the constructs (i.e., burnout and work engagement) as part of a system that assumes reciprocal relationships between variables, which could uncover previously unknown patterns of relationships between variables. In the next section, we demonstrate this statistically, based on previous research in other psychological fields (i.e., intelligence), and scrutinise the benefits of the network model by employing the network model (and the latent models) in three samples from the US, the UK, and Norway. The approach is based on Kan et al. (2020) which demonstrated how a psychometric network analysis (and mutualism theory) outperformed the previously established *g* factor model, when they compared several latent variable models and the network model.

## 2 Method

### 2.1 Transparency and Openness

All code, additional analysis and research materials are available at [https://osf.io/wgdvh/]. The complete analysis code is based on previous work by Kan et al. (2020) and Skjerdingstad et al. (2022). Data were analysed using R, version 07.2 (R Core Team 2022) and the Psychonetrics' (version 0.10) package default settings. This study's design and its analysis were not preregistered.

### 2.2 Sample and Data Collection

The data in this study were collected in Norway, the US and the UK. The first estimation was carried out on an open dataset available from the University of South‐Eastern Norway Archive (Øvergård et al. 2022). The cross‐sectional dataset was formerly used by Gottenborg et al. (2022) to examine the nomological validity and psychometric properties of a People Performance Scales. People Performance Scale is a research‐based survey instrument that measures work environment characteristics across 15 measurement scales. The data was gathered by an online survey in two large organisations (a governmental agency and a worker's union) in Norway. A total of 2819 respondents was invited to answer the survey. The final sample consisted of 2469 respondents (87% response rate). Of these respondents, 2306 (93.4%) were from the governmental agency, while 163 (6.6%) were from the worker's union. We removed an additional five respondents because of missing data. The sample consisted of 58.2% females and ranged from 19 to 72 years of age (*Mean* = 46.9, *SD* = 10.60). Employees was the majority in the sample (88.6%) while leaders represented the minority (11.4%).

The second estimation was carried out on a cross‐sectional sample of employees from the US and the UK, gathered for the purpose of this study. A short questionnaire was developed based on the items used to measure burnout and work engagement in the Norwegian sample. The questionnaire was sent to 1006 employees using Prolific, a subject pool for online data gathering (Palan and Schitter 2018). The biggest representation was from health care (14.1%), education (13.3%) and retail (11%). 49.4% of the sample was female and the age ranged from 18 to 65 or older (*Mean =* 39.57, *SD =* 13.12). To increase the robustness, reproducibility, and trustworthiness of the sample, we used Aguinis, Villamor, and Ramani (2021) guidelines for data gathering and analysis using subject pool data. We used Prolific ID check, attention checks, and only English‐speaking countries to ensure data validity (Aguinis, Villamor, and Ramani 2021). We removed 12.62% (127 participants) due to failed attention checks. The final sample consisted of 879 employees from different industries.

The decision to combine the sample from the US and UK was based on theoretical and practical reasons. Firstly, we expect (and test) cross‐cultural validity of the relationship between burnout and work engagement (Dong and Dumas 2020; Leitgöb et al. 2023). Secondly, higher burnout and lower work engagement seem to be more prevalent in countries where they expect and accept power to be distributed unequally, and in countries where they value career success over quality of life (i.e., the US and UK; House et al. 2004; Schaufeli 2017a, 2017b). While the three countries share certain features such as being considered WEIRD (Western, Educated, Industrialised, Rich, and Democratic; Henrich, Heine, and Norenzayan 2010), and score similarly on in‐group collectivism and human orientation, the US and UK could be considered similar to each other, and slightly different from Norway. The US and UK score high on performance orientation, are clustered as Anglo‐countries, share history of migration, and share ethnic and linguistic similarities (House et al. 2004). Thus, they share several characteristics that may influence the interpretation of items. Norway, on the other hand, is considered part of the Nordic countries which score lower on performance orientation and higher on gender egalitarianism, institutional collectivism, and uncertainty avoidance (House et al. 2004). This could reflect a cultural preference for cooperation over competition. Finally, the US and UK sample was combined due to practical reason. The data collection only gathered responses from 48 respondents from the US, with the remaining 958 from the UK, which would lead to difficulties in the statistical analysis.

### 2.3 Measures

#### 2.3.1 Burnout

Burnout was measured with an exhaustion and a cynicism scale in the Norwegian sample and the UK/US sample. The exhaustion scale consisted of four items from (Pejtersen et al. 2010). One sample item is: ‘I often feel exhausted’. The cynicism scale was measured with three items from Bang and Reio Jr (2017). One sample item is: ‘I have become less enthusiastic about my work’. The reliability of the scale was estimated by the omega (*ω*) coefficient and found to be satisfactory in the Norwegian sample (*ω* = 0.88) and the US/UK sample (*ω* = 0.89). All items were in a five‐point Likert format, ranging from ‘Fully disagree’ (1) to ‘Fully agree’ (5).

#### 2.3.2 Work Engagement

Work engagement was measured with a short and a long version of UWES (Schaufeli, Bakker, and Salanova 2006). In the Norwegian sample, work engagement was measured with six items. In the US and UK sample, work engagement was measured with the original nine items from UWES (Schaufeli, Bakker, and Salanova 2006). The reliability of the scale was estimated by the omega (*ω*) coefficient and found to be satisfactory in the Norwegian sample (*ω* = 0.90) and in the US/UK sample (*ω* = 0.92). One sample item is: ‘At my work, I feel bursting with energy’. The response format was a five‐point Likert scale ranging from ‘Fully disagree’ (1) to ‘Fully agree’ (5).

### 2.4 Statistical Analyses

Before estimating the factor and network models, a preliminary analysis was carried out. First, the measurement invariance (i.e., differences in item means between countries) was examined. Then, the possibility of common method variance was examined. To examine the differences in means, ANOVA with the item as a dependent variable and country ID as an independent variable was carried out. To examine the potential for common method variance, a Harmann's single factor test with Maximum Likelihood estimation was carried out.

To compare the factor and network models, we followed the procedure of Kan et al. (2020). The comparison between factor and network models was considered to answer which of the models provides the best explanation of the variance‐covariance structure among the observed variables. Since a full network model is not constrained this may not be interpreted as the most interesting from a theoretical perspective. However, the network model can be considered the null‐model against which factor models are tested (Kan, van der Maas, and Levine 2019). We then examined the network structure of burnout and work engagement to determine node and bridge centrality. This provides additional understanding about the relationship between burnout and work engagement, particularly concerning which nodes are most central in connecting the identified communities in the network (Jones, Ma, and McNally 2021).

**FIGURE 1:**
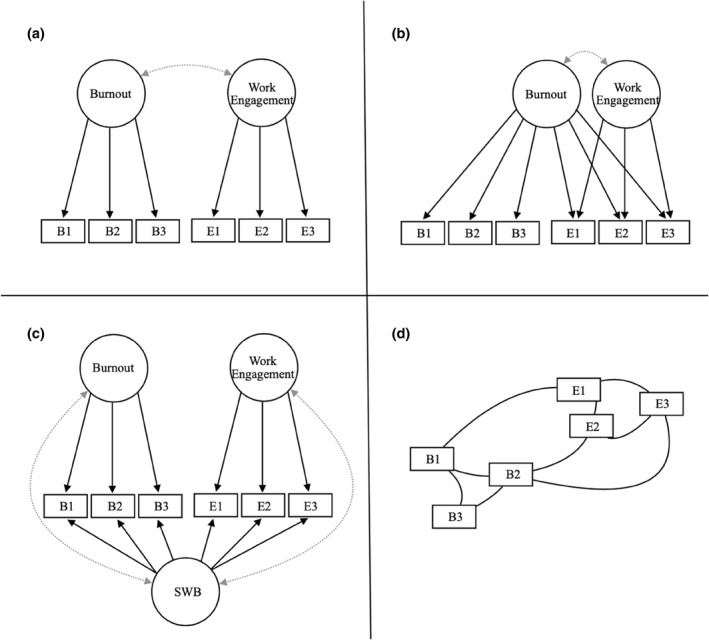
The theoretical models examined in this study across samples from the US, UK, and Norway. Three hypothetical items (B1‐B3, E1‐3) from each construct is displayed. SWB, subjective well‐being. The four models was (a) a distinct (but related) factor model, (b) a continuum factor model, (c) a dialectical bifactor model, and (d) a dialectical network model.

We estimated four models displayed in Figure 1. The four models were (a) a distinct (but related) factor model, (b) a continuum factor model, (c) a dialectical bifactor model, and (d) a dialectical network model. The ‘distinct but related’‐model was based on Schaufeli et al. (2002). In this model, exhaustion and cynicism are defined as facet variables of a latent burnout construct, while vigour, dedication and absorption are facet variables of a latent work engagement construct. The ‘continuum’‐model was based on the final model in the meta‐analysis by Cole et al. (2012). In their study, burnout is the latent factor for all burnout and work engagement variables, while work engagement is a latent factor only for vigour, dedication, and absorption variables. The last factor model, the ‘dialectical bifactor model’, was based on the recent paper by Trógolo et al. (2020). We examine if a general well‐being construct with all variables loading on a general factor is possible, given the measurements and the inclusion of absorption. This means that burnout and work engagement load on each of their respective variables as well. Finally, the estimated network model was calculated with the full partial correlation matrix and then pruned (*α* = 0.01). The Gaussian Graphical model was used to estimate the network structures using the R package *Psychonetrics* (Epskamp 2020). Nodes represent each of the variables in burnout and work engagement. The edges between the nodes represent partial correlation between variables when all other variables were held constant (Borsboom et al. 2021). Maximum likelihood estimation is used to obtain unbiased estimates of the parameters (Myung 2003).

The network and factor models were evaluated according to their absolute and relative fit by following standard evaluation criteria (Hu and Bentler 1999; Schermelleh‐Engel, Moosbrugger, and Müller 2003). TLI and CFI values ≥ 0.95 were considered indications of good model fit. RMSEA values ≤ 0.06 were considered as indications of good absolute fit. The model with the lowest AIC and BIC values was considered to provide the best relative fit and the best summary of the data. According to Raftery (1995) a difference in BIC values between 6 and 10 is considered a meaningful difference between models, and beyond 10 is considered very strong.

Before creating the network models, we ensured that none of the variables included in the network overlapped conceptually by applying a consensus procedure and a data‐driven method (Fried and Cramer 2017; Jones, Ma, and McNally 2021). The correlation matrix was examined for linear combinations among variables and confirmed as positive definite (Blanchard et al. 2021). The goldbricker function was applied to identify redundant variables (Jones, Ma, and McNally 2021). Finally, dependent correlations were examined. Nonparanormal transformations were applied to deal with skewed data using the R package *huge* (Jiang et al. 2019). To visualise the network, the Fruchterman–Reingold algorithm was used, with nodes with the highest centrality being drawn to the centre of the network, and less important nodes are placed in the periphery. This algorithm also functions to minimise the number of crossing edges (Fruchterman and Reingold 1991). In addition, we obtained the centrality and bridge centrality indices for the variables in the two networks to provide additional information about their psychometric properties (Jones, Ma, and McNally 2021). Expected influence centrality is calculated with a standardised z‐score on the *x*‐axis. Expected influence reflect overall importance of a node in the network (low z‐scores corresponds to low importance of the node in the network), accounting for the presence of negative edges (Robinaugh, Millner, and McNally 2016). The bridge centrality indices displayed the most central nodes in bridging identified communities in the network (Jones, Ma, and McNally 2021).

## 3 Results

### 3.1 Preliminary Analysis for the Network Estimation

The ANOVA reveal significant mean differences between the US/UK sample and the Norwegian sample. For the Norwegian sample, the mean was higher for the engagement variables (> 4.0) and lower for the burnout variables (< 3.0). For the US and UK sample, there was no significant difference in means between the two English‐speaking samples, but, as mentioned, they both were significantly different from the Norwegian sample. Judging from the items, the respondents from the US and UK experienced more burnout and less engagement, generally averaging from 2.65 to 3.72 on all items. The Harmann's single factor test was acceptable and below the recommended threshold in the Norwegian (31%) and the US/UK sample (42%), which indicated that common method variance was not problematic.

The results from goldbricker analysis reported the presence of the following pairs of topologically overlapping variables in the UK/US sample: ‘I often feel physically exhausted’ (EX2) and ‘I often feel emotionally exhausted’ (EX3), and ‘I have become less interested in my work since I started in this job’ (C1) and ‘I have become less enthusiastic about my work’ (C2), and ‘At my job, I feel strong and vigorous’ (E9) and ‘I feel happy when I am working intensely’(E5). This empirical approach informs about the importance of removing one redundant variable in each identified redundant pair before continuing with the network analysis. The reason can be attributed to several factors such as a difference in how the variables were interpreted or applied within the cultural context, or possibly because of the initial item selection and validation that were more suited to the Norwegian context.

In the exhaustion pair, we removed EX2 based on the vast array of studies on burnout that show emotional exhaustion as impediment to our understanding of burnout (Bakker and Demerouti 2017). In the cynicism pair, we removed C2 because enthusiasm has a stronger positive connotation compared to C1. We removed E9 ‘At my job, I feel strong and vigorous’ because E5 was included in the first dataset, and we wish to compare the relationships in the network. In the Norwegian sample, the consensus procedure and the data‐driven method supported the theoretical validity of the variables, hence none of the variables were removed due to redundancy.

### 3.2 Comparing Network and Factor Models

The absolute and relative fit measures of the models are shown in Table 1 (Norwegian sample) and Table 2 (US and UK sample). According to fit criteria the network models are favoured over the theoretical, measurement and bifactor model in both networks. The network model has good absolute fit in the Norwegian sample (χ^2^(42) = 121.37, *p* < 0.001; CFI = 1.0, TLI = 0.99; RMSEA = 0.028) and in the US and UK sample (χ^2^(64) = 279.36, *p* < 0.001; CFI = 0.97, TLI = 0.96; RMSEA = 0.062). The network model has better relative fit compared to the factor models as indicated by ΔAIC (≥ 1403.94) and ΔBIC (≥ 1363.27) in the Norwegian sample, and ΔAIC (≥ 7370.72) and ΔBIC (≥ 7432.83) in the US and UK sample. The AIC and BIC values indicates that the network models provide the best representation of the phenomena by satisfying the criteria for a very strong difference between the models (Raftery 1995).

**TABLE 1:** Results of the model comparisons based on analyses in software package Pscyhonetrics using the Norwegian sample.

Model	*χ* ^2^(df)	*p*‐value	CFI	TLI	RMSEA [CI_90_]	AIC	BIC
NM	121.37 (42)	< 0.001	1.0	0.99	0.028 [0.022–0.034]	68,324.87	68,685.06
BFM	665.69 (52)	< 0.001	0.98	0.97	0.063 [0.058–0.068]	69,728.81	70,048.33
CM	5025.26 (58)	< 0.001	0.79	0.71	0.19 [0.18–0.19]	74,204.04	74,471.28
DM	5250.26 (64)	< 0.001	0.78	0.73	0.18 [0.18–0.19]	74,417.34	74,649.72

Abbreviations: BFM, Bifactor model; CM, Continuum model; DM, Distinct (but related) model; NM, Network model.

**TABLE 2:** Results of the model comparisons based on analyses in software package Pscyhonetrics in the US and UK sample.

Model	*χ* ^2^(df)	*p*‐value	CFI	TLI	RMSEA [CI_90_]	AIC	BIC
NM	193.87 (50)	< 0.001	0.98	0.97	0.057 [0.049–0.066]	25,083.53	25,341.52
BFM	489.25 (85)	< 0.001	0.96	0.95	0.072 [0.066–0.079]	32,454.25	32,774.35
CM	2296.63 (94)	< 0.001	0.80	0.75	0.16 [0.16–0.17]	34,214.57	34,491.68
DM	2404.63 (103)	< 0.001	0.80	0.76	0.16 [0.15–0.16]	34,256.13	34,490.25

Abbreviations: BFM, Bifactor model; CM, Continuum model; DM, Distinct (but related) model; NM, Network model.

### 3.3 The Network Model

**FIGURE 2:**
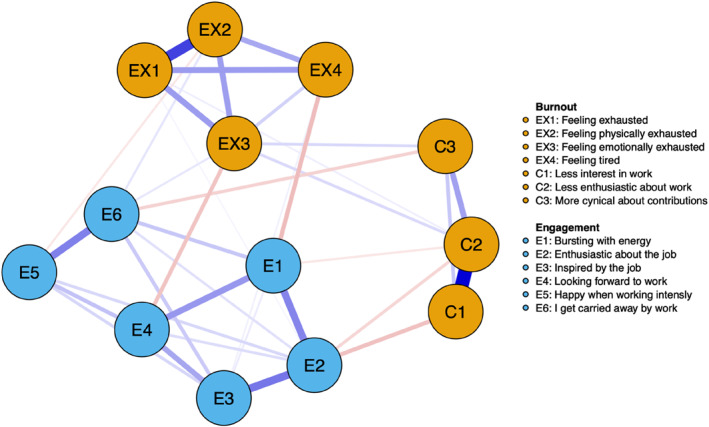
Network structure of burnout and work engagement in the Norwegian sample. The blue edges represent positive partial correlations between nodes and the red edges represent negative partial correlations.

**FIGURE 3:**
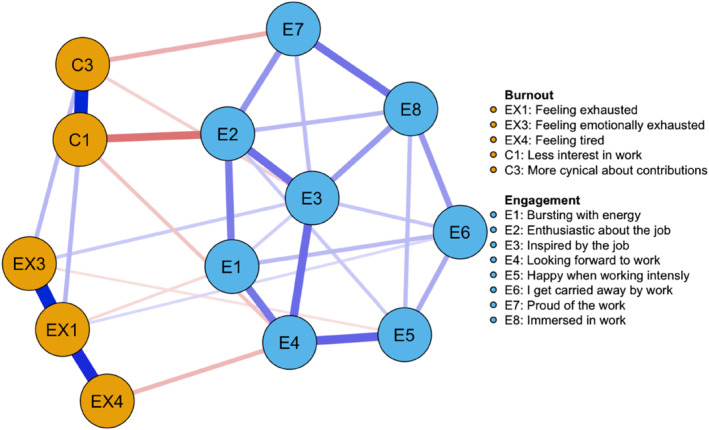
Network structure of burnout and work engagement in the US and UK sample. The blue edges represent positive partial correlations between nodes and the red edges represent negative partial correlations.

The network models are represented graphically in Figures 2 and 3. The two networks share several similarities, notably, there is a separation of three clusters (communities), one for work engagement, one for cynicism and one for exhaustion. The strongest relationships are between variables within the respective communities. In both networks, there are several negative but also a few positive relationships. In the Norwegian sample, negative connections exist between, firstly, being carried away by work (E6) and more cynical about contributions (C3), secondly, bursting with energy (E1) and feeling tired (EX4), and thirdly, being inspired by the job (E3) and showing less interest in work (C1). In the UK and US sample, negative connections are found between the following pairs: looking forward to work (E4) and feeling exhausted (EX4), enthusiasm about the job (E2) and less interest in work (C1), and finally, being proud of the work (E7) and being more cynical about its contributions (C3). Interestingly, the positive relationships between the burnout and work engagement communities are largely shared across the two networks. As shown in Figures 2 and 3, getting carried away by work (E6) and being inspired by the job (E3) share positive connections with feeling exhausted (EX1), feeling physically (EX2) and emotionally (EX3) exhausted, and feeling tired (EX4).

**FIGURE 4:**
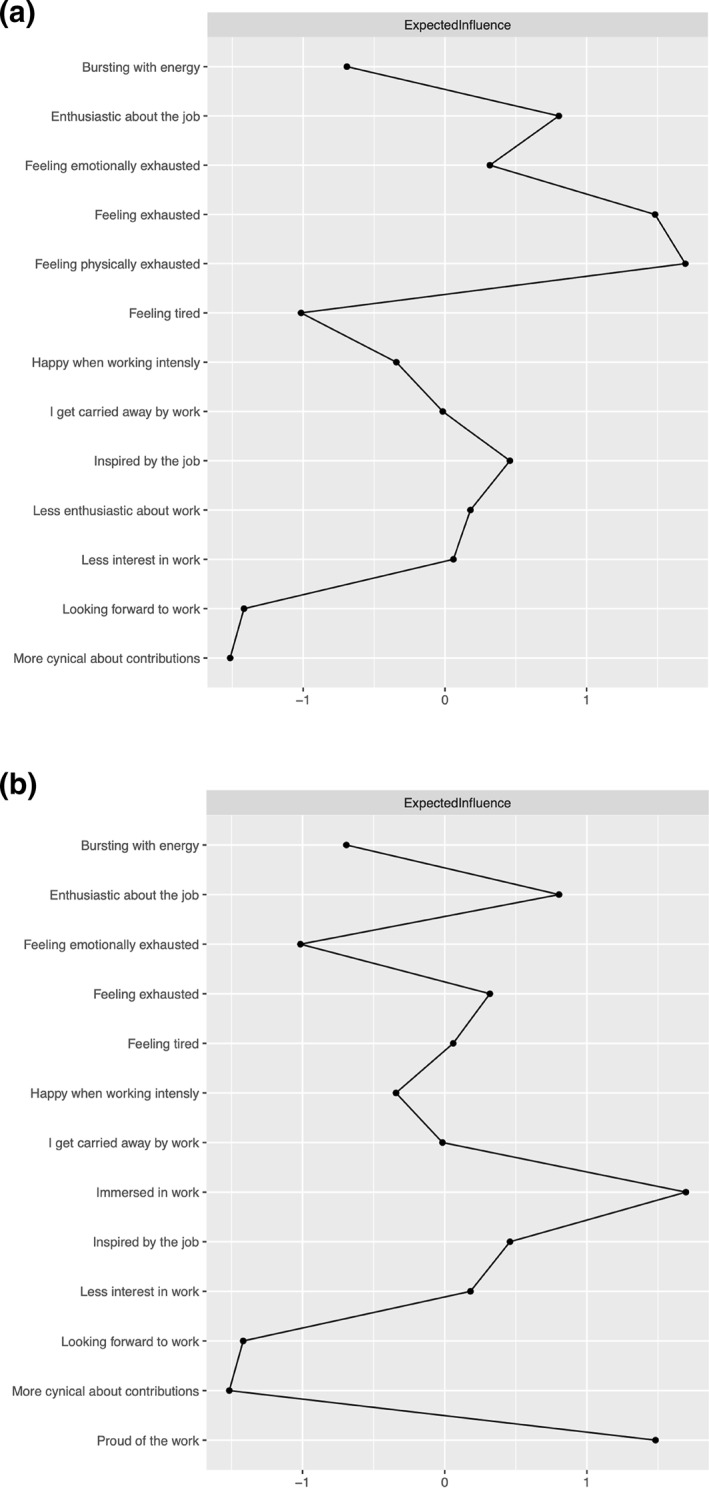
Expected influence estimates of the network. (a) The expected influence of the variables in the Norwegian sample network. (b) The expected influence of the variables in the US and UK sample network.

**FIGURE 5:**
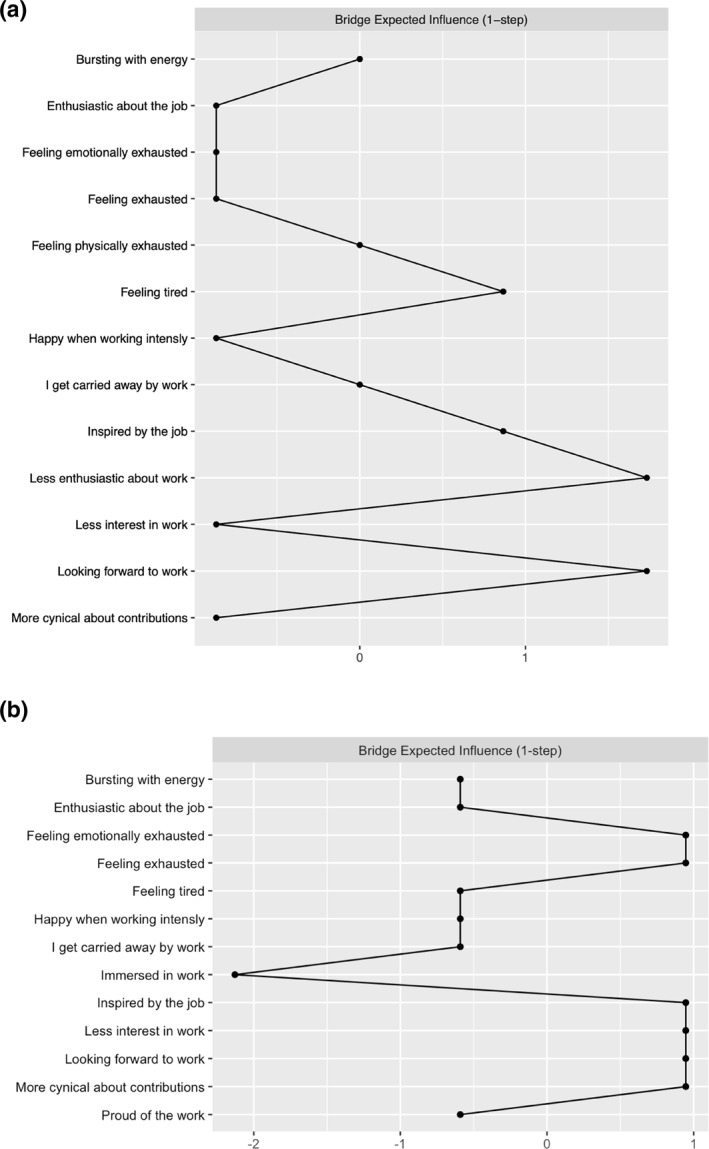
Bridge expected influence estimates of the networks. (a) The bridge expected influence of the variables in the Norwegian sample network. (b) The bridge expected influence of the variables in the US and UK sample network.

Figures 4 and 5 reveal which nodes have higher expected influence in the two networks and which nodes are likely to act as bridges. Centrality estimates are converted to standardised z‐scores on the *x*‐axis, where higher z‐scores correspond to higher importance of the node in the network, and lower z‐scores corresponds to low importance of the node in the network. A z‐score is a way of transforming a variable so that it has a mean of 0 and a standard deviation of 1. This standardisation allows for comparison between different variables that originally have different scales. In this context, a higher z‐score of 1 means that the variable (i.e. node) has a centrality or importance of one standard deviation above the mean. A z‐score of 0 reflect an average level of centrality or importance. A z‐score of −1 reflect that a node has centrality score or importance of one standard deviation below the mean (or average centrality). Bridge centrality provides us with information about which variables from one community (e.g. engagement) is most influential in its connectivity/association with variables from another community (e.g. burnout).

In the Norwegian sample, feeling exhausted (EX1) and feeling physically exhausted (EX2) have the highest influence estimates in the network. However, looking forward to work (E4) and being less enthusiastic about work (C2) are, as shown Figure 5 a and b, likely the bridge between cynical attitudes towards work, exhaustion, and work engagement. In the US and UK sample, feeling exhausted (EX1) and being inspired by the job (E3) have the highest influence estimates in the network. There are also several bridges that influence connectivity between the constructs in the network. For example, the variables that measure burnout, feeling exhausted (EX1), emotionally exhausted (EX3), less interest in work (C1), and more cynical about contributions (C3) are all important bridges in this network. While for work engagement, being inspired by the job (E3) and looking forward to work (E4) showed the highest bridge influence.

## 4 Discussion

The debate surrounding the distinctiveness of burnout and work engagement has undergone extensive empirical scrutiny over the past two decades. While Maslach, Jackson, and Leiter (1997) argued that work engagement was redundant, Schaufeli et al. (2002) argued that although *they may be* conceptually opposites, a separate measure of work engagement is necessary. This ultimately led to the notion of a dialectical relationship, as theorised by Leon, Halbesleben, and Paustian‐Underdahl (2015). However, this relationship has primarily been examined using latent variable modelling approaches (e.g., Taris, Ybema, and van Beek 2017; Trógolo et al. 2020). Furthermore, until now network modelling's usefulness in explaining this dialectical relationship has not been explored.

Our findings demonstrate that a network approach provides a suitable explanation of the relationship between burnout and work engagement across countries (Dong and Dumas 2020; Leitgöb et al. 2023), supported by theory (Leon, Halbesleben, and Paustian‐Underdahl 2015) and all fit indices (Hu and Bentler 1999; Raftery 1995; Schermelleh‐Engel, Moosbrugger, and Müller 2003). The network analysis reveals that burnout and work engagement share an intricate relationship. Although most relationships in the networks are negative, we observed positive associations between exhaustion variables and being inspired by the job and getting carried away by work. This suggests that the positive aspects of one's job, such as a state of high inspiration, can also be positively related to burnout. Our analysis further identifies three communities: burnout is separated into a community of cynicism and a community of exhaustion, while work engagement is one large community. Consequently, our study challenges the exclusion of the absorption component when examining the relationship between burnout and work engagement (e.g., Trógolo et al. 2020) and supports the original conceptualisation (Schaufeli 2012; Schaufeli et al. 2002; Seppälä et al. 2009). Taken together, these findings have several theoretical, empirical, and practical implications.

### 4.1 Theoretical Implications

From a theoretical perspective the network approach adds to our understanding of the potential dialectical relationship between burnout and work engagement (Leon, Halbesleben, and Paustian‐Underdahl 2015). Dialectical theory emphasises that a state of contradiction may arise when two seemingly opposing constructs are interdependent and negatively related (Baxter 1990; Leon, Halbesleben, and Paustian‐Underdahl 2015). When we contrast the network model with factor models, we obtain deeper insights into the merits of representing the dialectical relationship through network analysis. Although the network model was not strictly confirmatory, the networks in the two samples served as null models for the confirmatory factor models (Kan, van der Maas, and Levine 2019).

The credibility of the network model is bolstered by the many similarities in the two networks, which were tested across the US, the UK, and Norway. For example, burnout and work engagement predominately exhibited negative relationships, and exhaustion was influential in both networks. This indicate that each of these constructs counteract one another (Leon, Halbesleben, and Paustian‐Underdahl 2015). Moreover, the two estimated networks show initial support for the notion that burnout and work engagement act on each other (Leon, Halbesleben, and Paustian‐Underdahl 2015), through the positive relationship between being inspired and carried away by work and exhaustion, in both networks.

This study could thus shed light on why previous studies have found positive associations between work engagement and negative outcomes, such as exhaustion (Junker et al. 2021) and workaholism (Schaufeli, Taris and Bakker 2008). Prior studies have explained these findings with how engaged individuals are less likely to take short breaks (Bakker and Oerlemans 2016), are more likely to work while sick (Kinman and Wray 2018), invest more cognitive, emotional, and physical energy into work (Rich, Lepine, and Crawford 2010), and spend longer hours at work (Schaufeli, Taris and Bakker 2008). While this may be reasons for *how* employees become exhausted, they do not provide reasons for *why* highly engaged employees work longer hours and with higher intensity. We speculate, based on our results, that highly engaged employees may work longer hours and devote more energy into work simply because they are highly inspired and become carried away by work.

Our study serves as a steppingstone for future research that aim to investigate the dialectical relationship further. While our study provides initial evidence for a dialectical relationship between burnout and work engagement, it does not evaluate dialectical theory's contradiction and process over time (Leon, Halbesleben, and Paustian‐Underdahl 2015). Future research should extend our findings by incorporating temporal elements to depict how ‘being inspired by work’ and ‘being carried away by work’ is associated with exhaustion. Additionally, future research could examine the dynamic relationship between burnout and work engagement; how burnout and work engagement act on, change, and oppose each other. One important caveat is that longitudinal network models may require 20 or more measurements to be stable models, especially if within‐person centring and sample means are used per person (Epskamp et al. 2018; Jordan, Winer, and Salem 2020).

Secondly, by using a more congruent model of the proposed dialectical relationship between burnout and work engagement, our study broadens the empirical approaches to burnout and work engagement (Cole et al. 2012; Schaufeli et al. 2002; Trógolo et al. 2020). The network model holds promise for theories such as the Job Demands‐Resources model (Bakker, Demerouti, and Sanz‐Vergel 2023; Demerouti et al. 2000) and the Effort‐Recovery model (Fostervold and Watten 2022; Meijman and Mulder 1998) which emphasise burnout and work engagement. While extensive evidence exists about the role of high job demands and low resources for burnout, a network approach can enhance our understanding of the specific nuances tied to job demands and their associations with burnout. For example, does it change our conclusions when we model the relationship as a network of variables between job demands (e.g., working hard), burnout (e.g., feeling tired) and work engagement (e.g., being immersed in work)? Previous latent variable models have not been able display these types of nuances when investigating the burnout‐work engagement relationship (Cole et al. 2012; Schaufeli et al. 2002; Trógolo et al. 2020).

Finally, following this explanation, we also provide insight to the central problem of optimal points of intervention or prevention of burnout (see Demerouti et al. 2021). Traditional intervention strategies typically emphasise stress reduction through relaxation, mindfulness, and cognitive behavioural therapy (Demerouti et al. 2021). The network approach, however, has been used to design optimal intervention strategies in, for example, depression (Bak et al. 2016; Fisher et al. 2017; Lutz et al. 2018; Ryan and Hamaker 2022). Our results identify ‘high inspiration’ as a potential mechanism between work engagement and burnout. With this knowledge researchers can develop hypotheses and test if interventions, such as leadership training or a health promotion strategies (see Knight, Patterson, and Dawson 2019 for an overview), *prevent* stress symptoms by intervening in individuals with high inspiration. However, we must emphasise that the role of high inspiration for exhaustion symptoms must be confirmed in other cross‐sectional, as well as longitudinal studies.

### 4.2 Practical Implications

The potential benefits tied to fostering engaged employees who experience less strain and exhaustion, that is, higher performance, better health, and more citizenship behaviours, are important for both researchers and practitioners (Bakker, Demerouti, and Sanz‐Vergel 2023). While work engagement is generally sought after, our study suggests that leaders and employees should practice additional awareness when they are heavily inspired and gets carried away by their work. These employees may be, in the long run, set up to experience more fatigue and burnout compared to less engaged employees. Thus, employees should monitor their experiences of exhaustion. However, we want to emphasise specifically leaders, and not the employees themselves, because employees who inherent these characteristics are likely not aware of the potential damage long term. Also, even if they might be, they may not always be able to stop themselves due to the perceived importance and enjoyment of work.

To address this, leaders can implement several practical strategies (see Bakker, Demerouti, and Sanz‐Vergel 2023, for an overview). For example, they could regularly monitor workload and stress levels, either through surveys or in person, or they can encourage breaks and time off, promoting a balanced approach to work. Also, leaders may facilitate or encourage proactive work behaviours such as job crafting and playful work design. Finally, they could provide a supportive psychosocial work environment where employees share and discuss their well‐being with colleagues.

### 4.3 Limitations

There are some limitations in our study of the relationship between burnout and work engagement. First, the data in this study is gathered by self‐report questionnaires which may introduce common method variance. Although several studies have indicated that common method variance may not be as troublesome as expected (e.g., Brannick et al. 2010; Conway and Lance 2010; Semmer, Zapf, and Greif 1996), and the Harmann's single factor test revealed acceptable levels, using more objective measures of work engagement and burnout in future research is warranted (Podsakoff et al. 2003). Second, the high correlations within the three variable pairs EX2 and EX3, C1 and C2, and E5 and E9, in the US and UK sample (indicated by less than a 25% difference in relationships) may not be similar in other populations. However, this study is strengthened by the cross‐country comparison and provides valuable information about the potential semantic overlap between variables within the measurement scale. With a factor analytical approach, this would have largely been ignored. Lastly, our study is cross‐sectional. While this study emphasises the importance of introducing a novel approach to the burnout‐work engagement relationship, it would be interesting to examine the relationship longitudinally. This, in turn, would help cross‐validate and identify the direction of the positive relationships between the two work engagement variables (being inspired and carried away by work) and the exhaustion community.

## 5 Conclusion

In conclusion, the present study presents a novel perspective and approach to burnout and work engagement as a system of interacting and reciprocal relationships. Our analyses and results support the network approach as the preferred model of the burnout‐work engagement relationship, in contrast to the latent variable models. Furthermore, our analyses reveal that while most of the variables in the work engagement community is negatively related to the two burnout communities (cynicism and exhaustion), being inspired and being carried away by work are positively related to exhaustion. This has not been previously identified using the latent variable approach. This indicates that future examinations of the relationship between burnout, work engagement, and other constructs may benefit by granular mechanistic insights through a systems‐based network approach.

## Conflicts of Interest

The authors declare no conflicts of interest.

## Data Availability

The data that support the findings of this study are openly available in University of South‐Eastern Norway at https://doi.org/10.23642/usn.13365587.v1.
